# Genome Sequence of *Candidatus* Riesia pediculischaeffi, Endosymbiont of Chimpanzee Lice, and Genomic Comparison of Recently Acquired Endosymbionts from Human and Chimpanzee Lice

**DOI:** 10.1534/g3.114.012567

**Published:** 2014-09-11

**Authors:** Bret M. Boyd, Julie M. Allen, Valérie de Crécy-Lagard, David L. Reed

**Affiliations:** * Florida Museum of Natural History, University of Florida, Gainesville, Florida 32611; † Genetics and Genomics Graduate Program, University of Florida, Gainesville, Florida 32610; ‡ Illinois Natural History Survey, University of Illinois at Urbana-Champaign, Champaign, Illinois 61820; § Department of Microbiology and Cell Science, University of Florida, Gainesville, Florida 32611

**Keywords:** gene loss, genome erosion, primary-endosymbiont, gamma-proteobacteria, Pediculus

## Abstract

The obligate-heritable endosymbionts of insects possess some of the smallest known bacterial genomes. This is likely due to loss of genomic material during symbiosis. The mode and rate of this erosion may change over evolutionary time: faster in newly formed associations and slower in long-established ones. The endosymbionts of human and anthropoid primate lice present a unique opportunity to study genome erosion in newly established (or young) symbionts. This is because we have a detailed phylogenetic history of these endosymbionts with divergence dates for closely related species. This allows for genome evolution to be studied in detail and rates of change to be estimated in a phylogenetic framework. Here, we sequenced the genome of the chimpanzee louse endosymbiont (*Candidatus* Riesia pediculischaeffi) and compared it with the closely related genome of the human body louse endosymbiont. From this comparison, we found evidence for recent genome erosion leading to gene loss in these endosymbionts. Although gene loss was detected, it was not significantly greater than in older endosymbionts from aphids and ants. Additionally, we searched for genes associated with B-vitamin synthesis in the two louse endosymbiont genomes because these endosymbionts are believed to synthesize essential B vitamins absent in the louse’s diet. All of the expected genes were present, except those involved in thiamin synthesis. We failed to find genes encoding for proteins involved in the biosynthesis of thiamin or any complete exogenous means of salvaging thiamin, suggesting there is an undescribed mechanism for the salvage of thiamin. Finally, genes encoding for the pantothenate *de novo* biosynthesis pathway were located on a plasmid in both taxa along with a heat shock protein. Movement of these genes onto a plasmid may be functionally and evolutionarily significant, potentially increasing production and guarding against the deleterious effects of mutation. These data add to a growing resource of obligate endosymbiont genomes and to our understanding of the rate and mode of genome erosion in obligate animal-associated bacteria. Ultimately sequencing additional louse p-endosymbiont genomes will provide a model system for studying genome evolution in obligate host associated bacteria.

Many insect species are engaged in symbiosis with intracellular microbial symbionts (Kikuchi 2009; Dale and Moran 2006; Gosalbes *et al.* 2010; Duron and Hurst 2013). In some cases microbial symbiosis has permitted insects to persist on specialized diets that are nutritionally incomplete. This is because the endosymbiont provides the insect with the metabolic capacity to synthesize vitamins and/or amino acids absent in their diet (Douglas 1989). Metabolic provisioning by endosymbionts likely facilitated the evolution and radiation of economically and medically important insect groups, including blood-feeding lice. These nutritional provisioning endosymbionts (called primary-endosymbionts, or p-endosymbionts), are obligate, bacteriome bound, and vertically inherited. Relationships between insects and p-endosymbionts are complex and ensure that endosymbionts are passed on to new generations (Bright and Bulgheresi 2010).

The parasitic lice of humans, chimpanzees, and gorillas possess p-endosymbionts belonging to the genus *Candidatus* Riesia (Sasaki-Fukatsu *et al.* 2006; Allen *et al.* 2007, 2009; Hypsa and Krizek 2007; Novakova *et al.* 2009). These p-endosymbionts are housed in a large bacteriome visible in the abdomen of these lice (Ries 1930; Buchner 1965; Eberle and Mclean 1982, 1983; Sasaki-Fukatsu *et al.* 2006; Perotti *et al.* 2007; Bright and Bulgheresi 2010). In sexually mature females, the p-endosymbionts leave the bacteriome and migrate to the ovaries, where they are passed on to the next generation of lice (Ries 1930; Buchner 1965; Eberle and Mclean 1982, 1983; Perotti *et al.* 2007; Bright and Bulgheresi 2010). Puchta (1955) conducted p-endosymbiont removal and louse-feeding experiments with human lice to determine whether p-endosymbionts provided lice with an essential compound absent in the louse’s diet (Perotti *et al.* 2009). Puchta found that the p-endosymbionts supplied the lice with seven different B vitamins (Puchta 1955 as interpreted by Perotti *et al.* 2009; Table 1). Loss of any of these vitamins reduced survival of louse nymphs (Perotti *et al.* 2009). One of these vitamins, vitamin B5 or pantothenate, had the greatest effect on louse survival when absent (Perotti *et al.* 2009). Pantothenate is the precursor to coenzyme A, and its synthesis by louse p-endosymbionts appears to be crucial to the survival of human lice. The genes encoding for proteins involved in pantothenate synthesis are located on a small plasmid (Kirkness *et al.* 2010) in *Candidatus* Riesia pediculicola. Localization of these genes on a plasmid could be significant in control and regulation of the pathway.

**Table 1:** B vitamins predicted to be supplied to human lice by their p-endosymbiont, *Ca*. Riesia pediculicola (based on Puchta 1955 as interpreted by Perotti *et al.* 2009), and if genes associated with vitamin synthesis where detected in p-endosybmiont genomes

B Vitamin	Effect on Louse if Vitamin Absent	Human Louse p-Endosymbiont	Chimpanzee Louse p-Endosymbiont
Thiamin (B1)	High female mortality, males survive to adult	No, transport present	No, transport present
Riboflavin (B2)	High mortality during second molt	Yes	Yes
Folic Acid (B9)	High mortality during second and third molts	Yes	Yes
Pyridoxine (B6)	High mortality during second molt	Yes	Yes
Nicotinamide (B3)	High mortality during first molt	Yes	Yes
Pantothenate (B5)	Near complete mortality during first molt	Yes, plasmid based	Yes, plasmid based
β-biotin (B7)	High mortality during first molt	Yes	Yes

The genomes of insect p-endosymbionts have been under intense study in regard to minimal genome requirements and genome erosion (Moran and Mira 2001; Silva *et al.* 2003; Gil *et al.* 2004; Delmotte *et al.* 2006; Moran *et al.* 2008; Gosalbes *et al.* 2010). This is because these p-endosymbionts possess some of the smallest known bacterial genomes but likely possessed much larger genomes before entering into obligate endosymbiosis that eroded during symbiosis (Moran and Mira 2001; Silva *et al.* 2003). Recently, Allen *et al.* (2009) calculated mutation rates in the 16SrRNA gene of insect p-endosymbionts (see also Allen *et al.* 2007). They found that p-endosymbionts acquired by insects less than 100 million years ago (mya) have a much greater mutation rate in this gene than in associations greater than 100 mya. Of these p-endosymbionts, *Ca*. Riesia p-endosymbiont of lice had the fastest mutation rate and is the youngest known insect p-endosymbiont association (the association began ~13-25mya; Figure 1). This suggests that in p-endosymbionts the mutation rate at this locus is not constant, but that mutations are occurring much more frequently in young p-endosymbionts and slowing as the symbioisis ages. These results lead to the question, does global genome erosion proceed in a similar manner, quickly reducing the genomes then dramatically slowing or is genome erosion a more continuous process with no major changes in rate? Burke and Moran (2011) looked at gene loss in the endosymbiont of Aphids, *Serratia symbiotica*. They found evidence of numerous pseudogenes and small genomic deletions that could effectively reduce the number of genes during genome erosion. *Ca*. Riesia presents a unique opportunity to look at genome erosion in a recently acquired, rapidly evolving insect p-endosymbiont. This is because *Ca*. Riesia is the only young rapidly evolving insect p-endosymbiont for which we have a detailed phylogenetic history with known divergence dates for each species (Allen *et al.* 2007, 2009).

**Figure 1:**
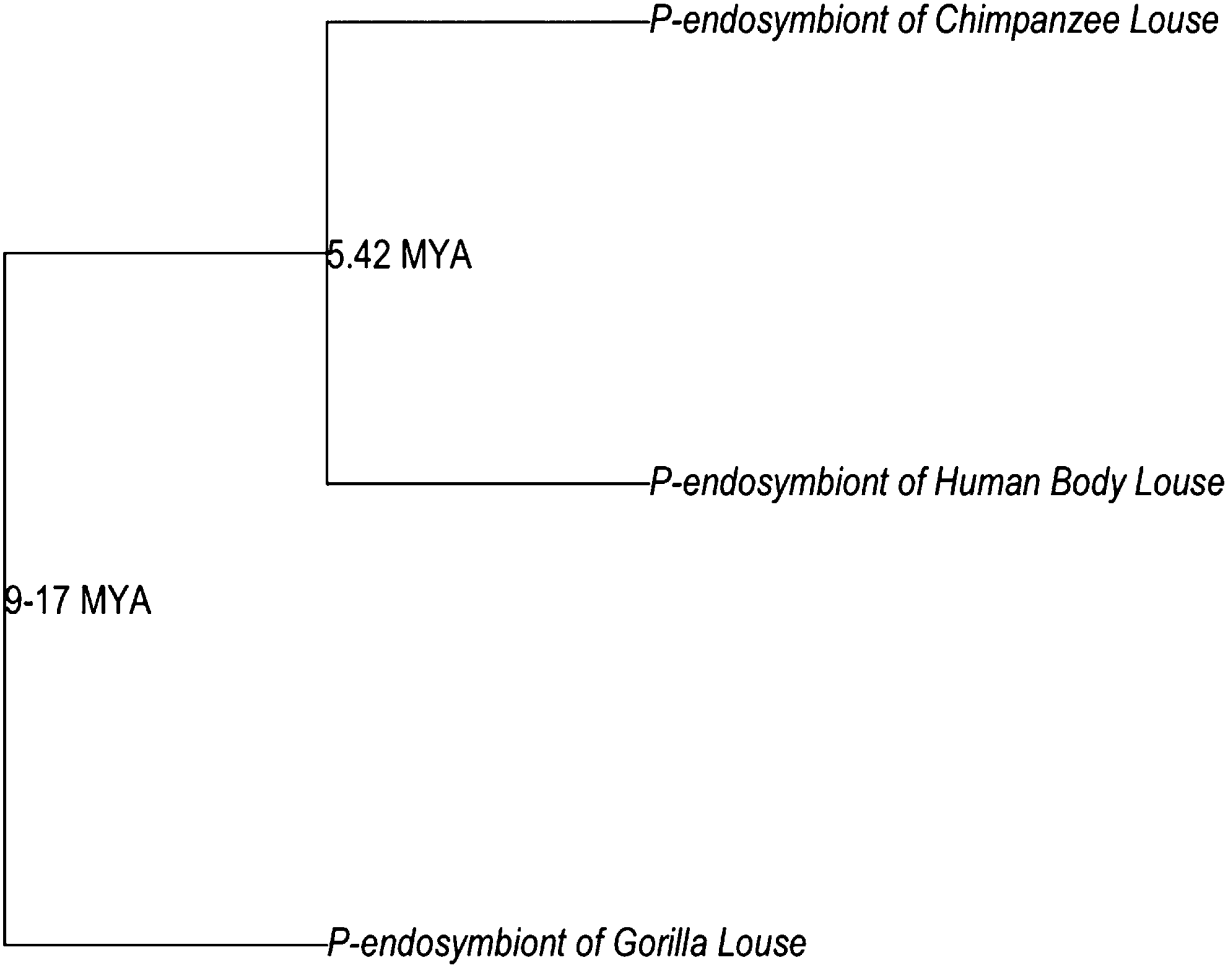
Evolutionary history of louse p-endosymbionts, *Ca*. Riesia species, with dates of species divergence. Origin of symbiosis at <25 million years ago and subsequent speciation (based on results by Allen *et al.* 2009).

Here we sequenced the genome of *Candidatus* Riesia pediculischaeffi, p-endosymbiont of chimpanzee lice, and compared its genome with that of the published genome of *Candidatus* Riesia pediculicola to estimate the rate of gene loss in louse p-endosymbionts. These two species diverged approximately 5.4 mya, only 7.6−19.6 million years after the symbiosis with lice began (Allen *et al.* 2009). Therefore, these species present an ideal opportunity to detect recent gene loss in young p-endosymbionts. We also found that these genomes contained numerous short-coding sequences of unknown function. Because we were interested in genome erosion leading to gene loss, we surveyed these short predicted coding sequences for conserved domains or other features that could describe their function. Finally, because these endosymbionts are believed to supply their hosts with B-vitamins absent in their diet we identified genes coding for proteins involved in B-vitamin synthesis to evaluate their roles as symbiotic bacteria.

## Materials and Methods

### Specimen collection

Specimens of *Pediculus schaeffi*, the chimpanzee louse, were collected from *Pan troglodytes schweinfurthii* (individuals Oketch and Ikuru) at the Ngamba Island sanctuary, Uganda. Specimens were stored in 95% ethanol and transported to the United States. From there they were stored in 95% ethanol at −80°.

#### Candidatus *Riesia pediculischaeffi str. PTSU genome sequencing*:

Genomic DNA was extracted from chimpanzee lice using a phenol-chloroform extraction method. Extracts from four lice were pooled, and a random shotgun library was constructed using Illumina’s TruSeq DNAseq Sample Prep Kit, selecting for an average insert size of 350 bp. The library was sequenced paired-end on the Illumina HiSeq2000 platform using the TruSeq SBS sequencing kit and analyzed using pipeline v.1.8 yielding 100bp reads. Quality of the read library was assessed using fastqc v.0.10.0 (Babraham Bioinformatics 2012).

### Assembly and annotation of the primary chromosome

We first removed repeat containing or simple sequence reads associated with louse telomeres to reduce the library complexity by excluding reads that mapped onto telomere scaffolds of the USDA strain Human Body Louse genome using Bowtie2 v.beta5 local alignment options (Langmead and Saizberg 2012). Remaining reads were trimmed to remove low-quality bases; removing 5 bases from the 5′ end, 7 bases from the 3′ end, and again trimming the 3′ end based on base call quality scores. If more than 25% of a read was removed by quality trimming, that read and its paired end mate, were removed from the library. Reads were then assembled *de novo* into contigs using Velvet v.1.2.02, kmer = 41 and long paired settings (Zerbino and Birney 2008; Zerbino 2010). Resulting contigs were compared with eight bacterial genomes (*Ca*. Riesia pediculicola USDA gi295698239, gi292493920; *Sodalis glossinidius* str. Morsitans gi85057978, gi85060411, gi85060466, gi85060490; *Wigglesworthia glossinidia* gi32490749, gi19225058, gi19225058; *Photorhabdus luminescens* subsp. Laumondii gi37524032; *Yersinia pestis* gi31795333; *Bacilus subtilis* subsp. subtiis gi223666304; *Buchnera aphidicola* str. APS gi15616630, gi10957103, gi10957099; and *Blochmannia floridanus* gi33519483) using NCBI-Blastn v.2.2.25 word-size = 11 (Altschul *et al.* 1990). These genomes were selected to be representative of a broad range of gamma-proteobacteria endosymbionts and a gram-positive species. Contigs with significant similarity to bacterial genomes were separated from the general population and considered as a tentative draft genome assembly of the p-endosymbiont. Bowtie2 v.beta5, end-to-end very-sensitive options, was used to build a library of reads aligning to the draft bacterial genome. The resulting population of reads was then reassembled *de novo* using both Velvet v.1.2.02 and ABySS v.1.3.4 into draft genome sequences (Simpson *et al.* 2009). The two draft sequences were compared with each other, and the ABySS assembly used more of the available reads and was selected as the final assembly. The draft genome sequence was annotated using the RAST pipeline (Aziz *et al.* 2008). The two genomes were compared using SEED individual metabolic pathway pages and sequence-based comparisons tools (Overbeek *et al.* 2005). Genes detected in the human louse p-endosymbiont genome but not in the chimpanzee louse genome were compared with the original population of contigs using blastn to search for and ensure no endosymbiont contigs were missed. The draft genome assembly of the chimpanzee louse p-endosymbiont genome was compared with the human louse p-endosymbiont genome using CoGe SynMap (Lyons *et al.* 2008; Lyons and Freeling 2008) to determine the order of contigs.

### Assembly and annotation of the pantothenate plasmid

A small plasmid was described by Kirkness *et al.* (2010) in the human louse p-endosymbiont that encodes proteins involved in pantothenate biosynthesis. A LAST (V.3; Frith *et al.* 2010) search was used to identify a homologous plasmid in the chimpanzee louse p-endosymbiont contig library (gi292493920). This search found matches to all three genes on a single contig in the initial chimpanzee louse contig library. Again, reads associated with this contig were isolated using bowtie2 v.beta6 and reassembled using ABySS v.1.3.4. Reads mapping to this contig were viewed in SAM format in Geneious (Biomaters; www.geneious.com) to determine that paired reads spanned the ends of the contig demonstrating that the assembled contig was circular. To annotate the chimpanzee louse p-endosymbiont plasmid, we extracted all open reading frames found in the plasmid. We then identified potential homologs by reciprocal tblastx best-hits between all chimpanzee louse p-endosymbiont plasmid open reading frames and predicted genes in the human louse p-endosymbiont plasmid. The plasmid sequence was then manually annotated based on reciprocal best-hit data, assigning the potential homologs to the predicted function given to the human louse p-endosymbiont plasmid.

### Estimating rates of gene loss in endosymbionts

Reciprocal tblastx was used to identify potential homologs in louse p-endosymbionts (perl scripts obtained from FAS Center for Systems Biology website were used to sort tblastx results for reciprocal best hits). Genes with reciprocal best hits were considered potential orthologs. If two genes in one genome were equally as good of a match for a gene in the other genome, both were retained as potential orthologs (this helped to correct for error when calculating rates of gene loss in louse symbionts by removing recently duplicated genes). We then identified genes for which an ortholog was not found. A manual check of genome alignments was then performed to determine whether any of these genes were actually present, but was not described in the genome annotation for any reason. These genes could then be considered lost in the other genome and rate of gene loss calculated using divergence dates described by Allen *et al.* (2009). Following Degnan *et al.* (2005) we calculated rate of gene loss as the absolute value of genes losses over the time since divergence.

### Identifying predicted genes of unknown function

The genome of the human louse p-endosymbiont is rich with short predicted coding sequences (CDS) of unknown function (Kirkness *et al.* 2010). We first wanted to determine if these hypothetical CDS possessed a shared conserved domain. To do this we first identified predicted CDS under 200 bp in length and were named hypothetical protein by the annotation pipeline. These predicted CDS were globally aligned to each other using muscle (implemented through Geneious). The alignments were then used to build a predicted conserved domain using hmmer V.3, hmmbuild (Eddy 2011). We then compared this conserved domain with the nonredundant protein and SWISS-PROT databases using hmmsearch (Eddy 2011) to determine whether it was similar to a known domain. The aforementioned process would be useful if these CDS proved to be mobile elements sharing a common history. If instead they were derived from different origins we would gain more information from comparing each individual gene to a database of conserved domains and protein sequences. Therefore, we compared all of the hypothetical CDS individually to the SWISS-PROT database using psi BLAST v.2.2.26 (Altschul *et al.* 1997).

### B-vitamin synthesis

We used the SEED database tools and blast searches to predict if the human and chimpanzee louse p-endosymbionts were capable of synthesizing B vitamins (Overbeek *et al.* 2005). Perotti *et al.* (2009), based on Puchta (1955), published a list of B-vitamins predicted to be provisioned to the louse by its bacterial p-endosymbiont. We accessed the SEED metabolic sub-system page for biosynthesis and metabolism for each of these B-vitamins using the seed viewer. From this predicted pathways we determined if predicted CDS in louse p-endosymbiont genomes supported synthesis of each B-vitamin.

## Results

### Chimpanzee louse p-endosymbiont genome assembly

Our genome assembly of *Ca*. Riesia pediculischaeffi resulted in reconstruction of a 576,757-bp primary chromosome (5 contigs, N50 = 303,941bp, GC = 31.79%) and a circular plasmid 5159 bp in length (1 contig). Depth of sequencing ranged from 120−150x coverage for the *Ca*. Riesia pediculischaeffi genome. The host organism (the chimpanzee louse) genome was sequenced at 50−100x coverage (see Johnson *et al.* 2014 for assembly of part of the louse genome using the same data set). Annotation of the primary chromosome resulted in 585 predicted coding sequences (herein referred to as genes) with five additional genes found on the plasmid. We did not find evidence of other bacterial genomes in our data.

**Figure 2:**
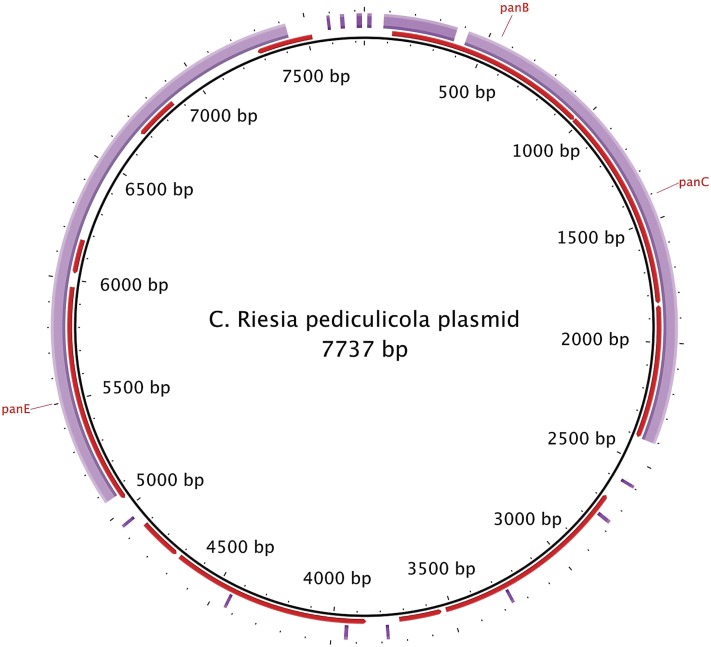
Alignment of the 5.2-kb plasmid from the chimpanzee louse p-endosymbiont to the 7.7-kb plasmid from the human louse p-endosymbiont that encodes genes involved pantothenate biosynthesis. Black inner ring is human louse p-endosymbiont plasmid reference sequence, red ring represents the annotation of human louse p-endosymbiont plasmid, and purple outer ring is alignment of query chimpanzee louse p-endosymbiont plasmid sequence. Genes involved in *de novo* synthesis of pantothenate (*panB*, *panC*, and *panE*) are labeled in red. Image generated using BRIG (Alikhan *et al.* 2011).

This genome was similar to the human louse p-endosymbiont (*Ca*. Riesia pediculicola) genome in size and composition (Table 2). Overall gene order and genomic synteny have been maintained between these species, with the exception of a duplicated region in the human louse p-endosymbiont at the end of the primary chromosome and a small duplicated region in the plasmid (Figure 2). These regions encode for duplicate copies of 11 genes.

**Table 2:** Genome and assembly statistics of Louse p-endosymbionts

	Human Louse p-Endosymbiont	Chimpanzee Louse p-Endosymbiont
Primary chromosome		
Number of bases	582127	576757
Number of contigs	n/a	5
Percent GC bases	28.57	31.79
Number of CDS total	556	585
Number of CDS unique	84	118
Pantothenate plasmid		
Number of bases	7737	5159
Number of contigs	n/a	1
Percent GC bases	35.25	37.1
Number of CDS total	12	5
Number of CDS unique	7	0

Human louse p-endosybmiont sequenced by Kirkness *et al.* (2010) Chimpanzee louse p-endosymbiont sequenced in this study. CDS total, sum of protein-coding sequences found in a given genome; CDS unique, sum of protein-coding sequences found in one louse p-endosymbiont genome, but not the other CDS, coding sequence.

Reciprocal tblastx searches revealed 472 potential orthologous CDS shared between the human and chimpanzee louse p-endosymbiont genomes. Additionally, 84 genes were predicted in the human louse p-endosymbiont genome not found in the chimpanzee louse p-endosymbiont genome. Ten of the genes unique to the human louse p-endosymbiont genome had a predicted function (Table 2). One hundred eighteen genes predicted in the chimpanzee louse p-endosymbiont genome were not found in the human louse p-endosymbiont genome. Fifteen of the genes unique to the chimpanzee louse p-endosymbiont genome had a predicted function (Table 1 and Table 2).

### Estimated rates of gene loss

We estimated rates of gene loss in louse p-endosymbionts when only considering genes with known function. The predicted rate of gene loss for the chimpanzee louse p-endosymbiont was 1.79 genes/million years (genes/my), whereas the rate for the human louse p-endosymbiont was 2.7 genes/my. If all unique genes (inclusive of small CDS of unknown function) are considered, then the rate increases to 14.29 genes/my for the chimpanzee louse p-endosymbiont and 20 genes/my for the human louse p-endosymbiont.

### Hypothetical short-coding sequences

Both louse p-endosymbiont genomes contain abundant short CDS with no known function. We failed to find a common conserved domain shared by most or all of these small CDS consistent with a phage or mobile element. We also failed to find significant similarity between individual short hypothetical CDS and any entry in the conserved protein databases. These sequences are prevalent throughout both endosymbiont genomes and are not restricted to any one location in the genome.

### B-vitamin synthesis

Genes associated with synthesis of the B vitamins riboflavin (B2), folate (B9), nicotinamide (B3), biotin (B7), and pyridoxine (B6) were found on the primary chromosome in both louse p-endosymbiont species (Table 1). Synthesis of riboflavin from GTP (*ribA*, *pyrD*, and *pyrR*) and transformation of riboflavin to FMN and FAD appears active. Metabolism from nicotinate to NADP+ is complete in both species. The pathway for folate contained the genes: *folE*, *folB*, *folK*, *folP*, *folC*, and a gene encoding for dihydrofolate-reductase was found in both taxa. In the human louse p-endosymbiont *folB* is duplicated, but not in the chimpanzee louse p-endosymbiont. Biosynthesis of biotin from PimeloylCoA appeared to be complete in both taxa [Biotin-protein ligase (EC6.3.4.15), *bioF*, *bioA*, *bioD*, and *bioB*]. *De novo* biosynthesis of pyridoxine phosphate appears complete in both taxa (*gapA*, *pdxB*, *pdxF*, *pdxA*, and *pdxJ*). In both taxa, synthesis of pantothenate (B5) was encoded by a small plasmid (*panE*, *panB*, and *panC*). In the chimpanzee louse p-endosymbiont, the *panE* gene, one of three genes involved in pantothenate biosynthesis, is truncated compared with the human louse p-endosymbiont. We failed to detect genes associated with thiamin (B1) biosynthesis; instead, we found genes encoding an ABC thiamin transport system that may act as part of an exogenous thiamin salvage in both louse p-endosymbionts (including a thiamin binding protein, a transmembrane component, and *thiQ* thiamin ATP binding protein).

## Discussion

### Genome structure

*Ca*. Riesia species possess small genomes (576,757 bp and 582,127 bp) with a low percent of GC bases (37% and 35%) that only encode for a few hundred (556 and 585) predicted protein-coding sequences. The human louse p-endosymbiont genome contains duplicated regions in both the end of the primary chromosome and on a small plasmid resulting in duplication of 11 genes. We did not find any evidence of these duplicated regions in the chimpanzee louse p-endosymbiont genome. By comparing only two genomes, we cannot determine whether these duplicated regions represent gains in the human louse p-endosymbiont genome or losses in chimpanzee louse p-endosymbiont genome. However, it seems parsimonious to conclude that they represent gains in the human louse p-endosymbiont genome because the duplicated genes are identical.

### Gene loss

Gene loss is important in shaping the genomes of insect p-endosymbionts (Moran and Mira 2001; Delmotte *et al.* 2006; Burke and Moran 2011). In young endosymbionts, genes unnecessary to maintain the symbiosis may be lost quickly. To determine whether whole gene loss was occurring in either or both louse p-endosymbiont genomes, we identified genes that were unique to each p-endosymbiont genome or present in both. We found that 202 of the predicted CDS were unique to one of the two louse p-endosymbiont genomes. This finding suggests that gene loss is occurring in these genome; however, we cannot differentiate between gene loss and addition when comparing two taxa. We have considered only loss for the purpose of this article. The number of unique genes was surprisingly high, but further investigation found that only a fraction of these predicted CDS showed homology to genes with known function from other bacterial genomes. Using a detailed phylogeny of human and primate louse p-endosymbionts with dates of speciation we were able to estimate rates of genes loss in these two p-endosymbionts.

When only those genes with a predicted function are considered, we find that the human louse p-endosymbiont is losing 2.7 genes/my and the chimpanzee louse p-endosymbiont is losing 1.79 genes/my. When similar methods are used, rates of gene loss have been reported for *Blochmannia floridanus* and *Blochmannia pennsylvanicus*, p-endosymbionts of carpenter ants that entered into symbiosis ~30 mya and diverged as distinct species ~16−20 mya by Degnan *et al.* (2005), which is slightly older than *Ca*. Riesia, the p-endosymbionts of lice. The ancestor of *Ca*. Riesia entered into symbiosis with a parasitic louse 13−25 mya and the p-endosymbionts of chimpanzee lice and human lice co-speciated with their hosts ~5.6 mya (Allen *et al.* 2009). Degnan *et al.* (2005) found that *B. floridanus* lost 1.56−1.25 genes/my (a rate similar to louse p-endosymbionts), but that *B. pennsylvanicus* lost genes at a much slower rate, 0.25−0.2 genes/my (see also Delmotte *et al.* 2006). Degnan *et al.* (2005) also reported rates of gene loss in *Buchnera* species at 0.6−0.42 genes/my (Table 3). *Buchnera* (p-endosymbionts of aphids) have been in association with aphids for >150 million years (Gosalbes *et al.* 2010), much longer than either *Blochmannia* or *Ca*. Riesia. *B. floridanus* and both *Ca*. Riesia species are losing genes at a faster rate than the ancient *Buchnera* p-endosymbiont, but *B. pennsylvanicus* showed the slowest rate of gene loss. Unexpectedly the younger p-endosymbiont *Ca*. Riesia is not losing genes at a faster rate than *B. floridanus*. The recently sequenced genome of *Blochmannia vafer* is smaller than either *B. floridanus* or *B. pennsylanicus* and may prove to have a faster rate of gene loss (Williams and Wernegreen 2010). Delmotte *et al.* (2006) interpreted this rate heterogeneity to mean that that rate of gene loss in insect p-endosymbionts is linage specific. We also see some heterogeneity in louse p-endosymbionts. However, additional sampling of other insect p-endosymbionts is needed to determine whether gene loss is truly lineage specific or if we can infer generalities about these rates.

**Table 3:** Age of associations between p-endosymbionts and insects and the estimated rate of gene loss in each p-endosymbiont

P-endosymbiont	Host Insect	Age of Symbiosis, my	Rate of gene loss, my
*Candidatus* Riesia pediculicola	Human lice	13−25	1gene/0.37
*Candidatus* Riesia pediculiscaeffi	Chimpanzee lice	13−25	1gene/0.56
*Blochmannia pennsylvanicus*	Carpenter ants	~30	1gene/4.0−50
*Blochmannia floridanus*	Carpenter ants	~30	1gene/0.64−0.80
*Buchnera aphidicola*	Aphid species	>150	1gene/1.70−2.38

Rates and ages for Blochmannia and Buchnera species from Degnan *et al.* (2005), ages of louse p-endosymbionts from Allen *et al.* (2009), and rates of gene loss in louse p-endosymbionts calculated in this study. my, million years.

### Abundant genes of unknown function

Both sequenced *Ca*. Riesia genomes possess abundant small (<200 bp) predicted genes of unknown function identified by the *ab initio* gene finder Glimmer (Delcher *et al.* 1999). Because one genome was sequenced using long-read technology and the other using short-read next-generation sequencing, we do not believe these short genes represent sequencing or assembly error (see Kirkness *et al.* 2010 for details on the assembly of the *Ca*. Riesia pediculicola genome). These genes are found in both *Ca*. Riesia genomes, but most genes are unique to either one genome or the other. This finding would suggest a rapid loss or expansion of these elements in each genome, consistent with a mobile element or phage. Mobile element activity could also help to explain the presence of duplicated regions in the human louse p-endosymbiont. If they are mobile elements or phage associated genes, then we should have detected similarities in overall nucleotide sequence or the presence of a conserved domain. Our searches failed to find evidence of conserved features. It is possible they represent an extinct mobile element or phage and that their structure has been disrupted by mutation. Sequencing of additional *Ca*. Riesia species that diverged earlier during the symbiosis, such as p-endosymbionts of Gorilla or human pubic lice (Allen *et al.* 2009), would allow us to differentiate between expansion or loss of these short genes. If expanding this would be indicative of a mobile element. Another possibility is that they represent degraded bacterial genes that are no longer identifiable. In this case we would be dramatically increasing the rate of gene loss in Ca. Riesia (1.79−2.7 genes/my to 14.29−20 gene/my). However, this must be interpreted with caution because short hypothetical genes may contribute little or nothing to a bacterial phenotype (Jackson *et al.* 2002, Lerat and Ochman 2005, Konstantinidis *et al.* 2006). Therefore, we might expect only the rate of genes loss for genes with a known role to represent gene loss impacting the bacterial phenotype.

### The role of *Ca*. Riesia as an endosymbiont

B vitamin synthesis is considered a primary function of p-endosymbionts for parasitic lice feeding on vertebrate blood (Puchta 1955; Perotti *et al.* 2009). Many of these vitamins are in low concentration or unavailable from the louses’ strict diet (Perotti *et al.* 2009). When human lice are treated to remove endosymbionts, the absence of different B vitamins had varying effects on louse survival and reproduction (Puchta 1955; Perotti *et al.* 2009). Here we found that the genomes of both human and chimpanzee p-endosymbionts encoded genes involved in the synthesis of these vitamins except for thiamin (Table 1). In both *Ca*. Riesia species, we found genes encoding for a mechanism to import thiamin across the p-endosymbiont cell membrane, the thiamin ABC transport. This is similar to an endosymbiont (*Sodalis glossinidius*) from the Tsetse fly, another blood feeding insect (Snyder *et al.* 2010). Here thiamin monophosphate is scavenged from exogenous sources by *Sodalis* using the thiamin ABC transport. The available exogenous source of thiamin is synthesized by a different endosymbiont, *Wigglesworthia glossinidia* (Snyder *et al.* 2010). It was surprising to see a similar mechanism in place for endogenous uptake of thiamin in p-endosybmionts of lice, as there are no other known endosymbionts present that could complement biosynthesis. Also, *Sodalis* possesses thiamin kinase and thiamin monophosphate kinase to convert the scavenged thiamin monophosphate to thiamin pyrophosphate that *Ca*. Riesia appears to lack. *Ca*. Riesia must possess an unknown kinase to salvage thiamin monophosphate.

*De novo* biosynthesis of pantothenate is considered to be the most important role of louse p-endosymbionts (Puchta 1955; Perotti *et al.* 2009). This pathway is encoded on a plasmid in both species. This plasmid may increase efficiency of production; particularly in the chimpanzee louse p-endosymbiont where one gene in the *de novo* pantothenate synthesis pathway is truncated.

Here we have sequenced the genome of a rare p-endosymbiotic bacterium, *Ca*. Riesia pediculischaeffi, found only in the parasitic lice of chimpanzees. Comparison of this genome with the genome of a closely related p-endosymbiont from human parasitic lice, *Ca*. Riesia pediculicola, revealed recent genome erosion and gene loss. Surprisingly this loss is not occurring significantly faster than in slightly older endosymbionts from ants, despite earlier evidence that the 16SrRNA genes in experiencing a higher mutation rate in louse p-endosymbionts (Allen *et al.* 2009). This genome sequence also revealed two surprises, an abundance of small genes of unknown function and the absence of genes for synthesis of vitamin B1 previously thought to be important to the symbiosis with lice. Additional sequencing of *Ca*. Riesia species would be significant to approach gene loss in this clade using a more rigorous phylogenetic framework employing ancestral state reconstruction.
